# Evaluation of the Effect of Systolic Blood Pressure and Pulse Pressure on Cognitive Function: The Women's Health and Aging Study II

**DOI:** 10.1371/journal.pone.0027976

**Published:** 2011-12-09

**Authors:** Sevil Yasar, Jean Y. Ko, Stephanie Nothelle, Michelle M. Mielke, Michelle C. Carlson

**Affiliations:** 1 Division of Geriatric Medicine and Gerontology, Department of Medicine, Johns Hopkins School of Medicine, Baltimore, Maryland, United States of America; 2 Center on Aging and Health, Johns Hopkins School of Medicine, Baltimore, Maryland, United States of America; 3 Department of Mental Health, Johns Hopkins Bloomberg School of Public Health, Baltimore, Maryland, United States of America; 4 Indiana University School of Medicine, Indianapolis, Indiana, United States of America; 5 Division of Epidemiology, Mayo Clinic, Rochester, Minnesota, United States of America; University of British Columbia, Canada

## Abstract

**Background:**

Evidence suggests that elevated systolic blood pressure (SBP) and pulse pressure (PP) in midlife is associated with increased risk for cognitive impairment later in life. There is mixed evidence regarding the effects of late life elevated SBP or PP on cognitive function, and limited information on the role of female gender.

**Methods/Principal Findings:**

Effects of SBPand PPon cognitive abilities at baseline and over a 9-year period were evaluated in 337 non-demented community-dwelling female participants over age 70 in the Women's Health and Aging Study II using logistic and Cox proportional hazards regression analyses. Participants aged 76–80 years with SBP≥160 mmHg or PP≥84 mmHg showed increased incidence of impairment on Trail Making Test-Part B (TMT, Part B), a measure of executive function, over time when compared to the control group that included participants with normal and pre-hypertensive SBP (<120 and 120–139 mmHg) or participants with low PP (<68 mmHg) (HR = 5.05 [95%CI = 1.42, 18.04], [HR = 5.12 [95%CI = 1.11; 23.62], respectively). Participants aged 70–75 years with PP≥71 mmHg had at least a two-fold higher incidence of impairment on HVLT-I, a measure of verbal learning, over time when compared to participants with low PP (<68 mmHg) (HR = 2.44 [95%CI = 1.11, 5.39]).

**Conclusions/Significance:**

Our data suggest that elevated SBP or PP in older non-demented women increases risk for late-life cognitive impairment and that PP could be used when assessing the risk for impairment in cognitive abilities. These results warrant further, larger studies to evaluate possible effects of elevated blood pressure in normal cognitive aging.

## Introduction

Hypertension (HTN), an elevation of systolic or diastolic or both systolic and diastolic blood pressures, is an important public health issue because of its high prevalence, approximately 26% in the general population [1], and because of associated high morbidity and mortality [2]. There is also evidence that age-related blood pressure changes occur and that these changes are gender-specific, namely systolic hypertension is more prevalent in elderly women than men [3]. These age-related blood pressure changes may account, in part, for the higher cardiovascular mortality reported among elderly females compared with elderly males [4]–[6], and should be considered an important target for preventive strategies in elderly females.

HTN has been extensively studied as a major risk factor for cognitive decline in older; community dwelling populations (see review [7]). While there is strong evidence that HTN in midlife is associated with increased risk for cognitive impairment later in life [8]–[13], there is mixed evidence regarding the effects of HTN in late life on cognitive function. One study reported no association [14], while other studies reported negative associations [15]–[18] between elevated baseline systolic blood pressure (SBP) and global cognitive function. Additionally, it is not clear whether specific cognitive abilities are more susceptible to the effects of elevated blood pressure in late life as one study showed a negative association between elevated SBP and learning and memory [19], another a negative association with attention, [20] and a third showed a negative association with naming and non-verbal memory [21]. There is currently no study available with a special focus on the role of SBP in late life and its effect on cognitive abilities in older women.

Pulse pressure (PP) is an independent predictor of all-cause and cardiovascular mortality [22]. High PP has been associated with Alzheimer's disease (AD) [23], poorer global cognitive function [24] and greater decline on verbal learning and non-verbal memory in individuals without dementia [25], when compared to participants with lower PP. However, there is currently no information available with a focus on the role of PP in late life and its effect on cognitive abilities in older women.

In the present study, we examined whether elevated SBP or PP in older women was associated with changes in cognitive abilities at baseline and over a 9-year period in a population of non-demented community-dwelling female participants, aged 70 to 80 years at baseline, in the Women's Health and Aging Study (WHAS) II.

## Methods

### Ethics Statement

This study was approved by the Johns Hopkins Institutional Review Board, and each participant gave informed, written consent before completing a standardized interview at each exam.

### Data Source and Study Population

This study involved secondary data analysis using the WHAS II, a prospective study of physical functioning among the least disabled two thirds of 70- to 80-year-old, community dwelling women in eastern Baltimore, MD. Sampling and recruitment of this cohort is described in detail elsewhere [26] and complements WHAS I, a study of the one-third most disabled, community-dwelling older women. Trained interviewers determined eligibility at sampling according to whether individuals were (a) aged 70–79 years; (b) had sufficient hearing and proficiency in English to be interviewed; (c) could be contacted by telephone; (d) had a Mini-Mental State Exam (MMSE) [27] score >24; and (e) reported difficulty in no more than one of four functional domains: mobility and exercise tolerance, upper extremity strength, higher functioning (e.g., shopping), and basic self-care. Of 880 eligible individuals, 436 agreed to participate in the baseline examination. Those agreeing to participate were more highly educated and had more diseases than those who refused, but did not differ in other characteristics. Five follow-up exams were conducted at approximately 1.5-year intervals, with the exception of a 3-year interval between Exams 3 and 4, yielding a maximum of 9 years of follow-up. Each follow-up visit was conducted in the clinic or home, as needed and included standardized physical, cognitive and functional evaluations, and collection of demographic, psychosocial, medical and medication information. Over 9 years, 90 participants died and 103 participants were lost to follow-up.

Individuals who were missing blood pressure information at baseline were excluded (N = 2). Individuals who had scores under the impairment threshold (described below) were later excluded for longitudinal analyses (N = 98). The 336 remaining individuals represent 77.3% of the 436 participating individuals, and were included in both the cross-sectional and longitudinal analyses. The 100 individuals excluded from this study were comparable to those included in the study on age, systolic or diastolic blood pressure, and history of hypertension, but had lower MMSE scores (p<0.001), lower education (p<0.001), and were more likely to be black or other race (p<0.001).

### Measures of Cognitive Function

Standardized cognitive testing by a trained technician was designed to comprehensively assess cognitive abilities in healthy older adults, and to be maximally sensitive to changes occurring with normal aging and to pathological changes occurring with dementia. Global cognitive status was assessed by the Mini-Mental State Exam (MMSE). The MMSE assesses five areas of cognitive function including orientation, attention, calculus, recall and language. The maximum score is 30 and a score of 24 or lower is suggestive of cognitive impairment. The Trail Making Test (TMT) [28], which is a pencil-and-paper test, was used to evaluate psychomotor speed via Part A and B, and executive function via Part B. Part A requires one to connect, as quickly as possible, a randomly distributed array of numbers sequentially from 1 to 25. Part B requires one to connect randomly distributed numbers and letters in an ascending alpha-numeric sequence. Participants were allotted a maximum time of 240 seconds on Part A and 360 seconds on Part B. Verbal immediate and delayed recall memory of 12 common objects were assessed using the Hopkins Verbal Learning Test-Revised (HVLT-R) [29]. Participants heard and recalled words during three successive learning trials (maximum = 36) and once after a 20-minute interval (maximum = 12).

Impairment on each of the cognitive assessments was defined as follows: Trail Making Part A (TMT-A) scores ≥81 seconds; Trail Making Part B (TMT-B) scores ≥225 seconds; Hopkins Verbal Learning Test immediate recall (HVLT-I) scores ≤16; Hopkins Verbal Learning Test delayed recall (HVLT-D) scores ≤4; MMSE scores ≤23 [30], [31]. These cut-points corresponded to approximately 1.5 to 1.8 standard deviations below internal norms at baseline on most tests.

### Measures of Blood Pressure

At each study visit, blood pressure (BP) was measured by a trained nursing staff according to protocols. The participant's BP was measured while the participant was resting and sitting in an upright position with legs uncrossed and feet flat on the floor for approximately five minutes. Three blood pressure readings, with 30-second intervals between each measurement, were obtained on the right arm using a mercury sphygmomanometer with an appropriate-sized occluding cuff and were averaged for data analysis.

Systolic hypertension status was stratified according to SBP readings as control group (SBP≤139 mmHg), HTN I group (SBP = 140–159 mmHg) and HTN II group (SBP≥160 mmHg) PP, was defined as the difference between SBP and DBP, and participants were assigned to the lower (PP = 48–68 mmHg), middle (PP = 71–77 mmHg) and upper (PP = 84–108 mmHg) tertile group according to their PP, where the lower tertile group served as the control group.

### Statistical Analyses

STATA 10.2 was used for all analyses (StataCorp, College Station, TX). We compared baseline demographic and health characteristics by HTN stage and PP tertile using chi-square tests for categorical variables and ANOVA with pair wise comparisons for continuous variables. In order to assess the baseline prevalence of impairment in cognitive abilities first cross-sectional analysis, using logistic regression models, were conducted. In order to assess the long-term effect of SBP and PP on the risk of developing impairment in each cognitive ability over the 9-year follow-up period, longitudinal, multivariate discrete-time Cox proportional hazard models [32] were conducted. The discrete-time Cox models have advantages over traditional, continuous proportional-Cox models in that the event, such as last visit or death, can occur over a discrete time; use of this model is especially appealing in analyzing longitudinal data with regularly scheduled visits. The model compares each case of newly diagnosed impairment with all other subjects in the study who were free of impairment at that visit when the impairment was diagnosed. Subjects contributed information up to the visit when the diagnosis of impairment occurred, or up to their last study visit.

Analyses, in Model 1 were first adjusted for the well known confounding effects of age [33], race (white vs. non-white) [34] and education (<12,  = 12, >12 years of education) [35]. Then, in Model 2, in addition to age, race, and education they were adjusted for income (<$10,000/yr, $10,000–24,999/yr, $25,000–49,999, >$50,000/yr), smoking status (never vs. ever), comorbidities such as history of HTN, stroke, myocardial infarction (MI) or angina, congestive heart failure (CHF), peripheral artery disease (PAD), diabetes mellitus (DM); depression (measured by Geriatric Depression Scale [36]), Body Mass Index (BMI, Body Mass Index (kg/m^2^), serum glucose level (mg/dl), total cholesterol level (mg/dl), and history of ever taking antihypertensive medications. The *a priori* p-value was set at p<0.05.

First, we evaluated the effect of SBP and PP on cognitive abilities in all participants, then, in separate analyses, we stratified subjects according to their age, 70–75 years old or 76–80 years old, to evaluate the possible role of age.

## Results

### Participants

The average age of the 336 participants at baseline was 74.1 years (ranging from 70–80 years), 41% had a college education and 81% were white (Table 1). 51% reported history of HTN and 52% reported antihypertensive medication use (Table 1). The prevalence of stroke, MI, angina, CHF, PAD, and DM, was 3.6%, 8.9%, 14.9%, 7.6%, 9.6% and 9.4%, respectively. The baseline means for MMSE, TMT Part A and Part B times (sec), and HVLT-I and -D scores were indicative of a high functioning sample (Table 2).

**Table 1:** Baseline Sociodemographic Characteristics of Participants by Hypertension Status and Pulse Pressure, WHAS II (N = 336).

Characteristics					PP	PP	PP	Statistics
	Control Group	HTN I	HTN II	Statistics	Lower Tertile	Middle Tertile	Upper Tertile	
	(N = 103)	(N = 124)	(N = 109)	*p*	(N = 117)	(N = 107)	(N = 112)	*p*
	N (%)				N (%)			
Age (Mean, SD)	73.5 (2.9)	73.7 (2.8)	74.1 (2.7)	0.2	73.4 (2.7)	73.9 (2.9)	74.02 (2.7)	0.2
Race:								
White	87 (84.5)	101 (81.5)	97 (89.0)	0.3	90 (76.9)	95 (88.8)	99 (88.4)	0.02
Education:								
<12 years	9 (8.7)	11 (8.9)	7 (6.4)	0.5	12 (10.3)	12 (11.2)	3 (2.7)	0.01
= 12 years	43 (41.7)	61 (49.2)	57 (52.3)		48 (41.0)	45 (42.1)	68 (60.7)	
>12 years	51 (49.5)	52 (41.9)	45 (41.3)		57 (48.7)	50 (46.7)	41 (36.6)	
Income ($/year):								
<10,000	20 (24.4)	25 (24.5)	21 (23.1)	0.5	24 (24.5)	24 (27.9)	18 (20.0)	0.6
10,000–24,999	27 (32.9)	32 (31.4)	38 (41.8)		33 (33.7)	26 (30.2)	38 (42.2)	
25,000–49,999	19 (23.2)	23 (22.6)	22 (24.2)		26 (26.5)	20 (23.3)	18 (20.0)	
>50,000	16 (19.5)	22 (21.6)	10 (11.0)		15 (15.3)	16 (18.6)	16 (17.8)	
Marital status:								
Married	64 (62.1)	70 (56.5)	70 (64.2)	0.4	77 (65.8)	60 (56.1)	68 (60.7)	0.3
Not Married	39 (37.9)	54 (43.6)	39 (35.8)		40 (34.2)	47 (43.9)	44 (39.3)	
Smoking:								
Never	57 (55.3)	67 (54.0)	57 (52.8)	0.91	65 (55.6)	59 (55.1)	58 (52.3)	0.9
Ever	46 (44.7)	57 (46.0)	51 (47.2)		52 (44.4)	48 (44.9)	53 (47.8)	
Hx HTN	29 (27.9)	68 (54.8)	75 (68.8)	<0.001	51 (43.6)	52 (48.6)	69 (61.6)	0.02
Hx Stroke	4 (3.9)	4 (3.2)	4 (3.7)	0.7	5 (4.3)	4 (3.7)	3 (2.7)	0.5
Hx MI	6 (5.8.2)	11 (8.9)	11(10.1)	0.5	5 (4.3)	7 (6.5)	16 (14.3)	0.02
Hx Angina	19 (8.3)	18 (14.5)	14 (12.8)	0.5	16 (13.7)	19 (17.6)	16 (14.3)	0.7
Hx CHF	6 (5.8)	8 (6.5)	9 (8.3)	0.8	10 (8.6)	2 (1.9)	11 (9.8)	0.04
Hx PAD	10 (9.6)	9 (7.3)	12 (11.0)	0.6	12 (10.3)	6 (5.6)	13 (11.6)	0.3
Hx DM	8 (7.7)	12 (9.7)	6 (5.5)	0.5	11 (9.4)	7 (6.5)	8 (7.1)	0.7
Ever taking HTN medications								
	54 (51.9)	101 (81.5)	92 (84.4)	<0.001	77 (65.8)	79 (73.8)	90 (80.4)	0.04

	Mean (SD)				Mean (SD)			
SBP (mmHg)	129.1 (9.1)	148.9 (5.0)	177.6 (12.0)	<0.001	138.1 (18.4)	150.6 (17.1)	167.9 (17.1)	<0.001
DBP (mmHg)	67.4 (10.1)	73.4 (11.8)	88.2 (17.0)	<0.001	80.0 (15.9)	76.6 (16.8)	72.1 (13.5)	<0.001
PP (mmHg)	61.7 (12.6)	75.5 (12.7)	89.5 (17.7)	<0.001	58.1 (10.0)	73.9 (3.4)	95.8 (12.0)	<0.001
BMI	25.9 (5.7)	27.3 (5.0)	26.5 (4.6)	0.1	26.7 (5.4)	26.2 (4.5)	27.0 (5.4)	0.5
GDS	3.7 (3.6)	3.9 (4.1)	3.5 (2.9)	0.7	3.4 (3.3)	4.2 (4.0)	3.7 (3.5)	0.3
Mean years to follow up								
	6.4 (8.7)	7.0 (6.0)	6.6 (6.2)	0.8	6.4 (8.2)	7.4 (4.9)	6.3 (7.3)	0.4

Control group (SBP≤139 mmHg); HTN I = Hypertension I (SBP 140–159 mmHg); HTN II = Hypertension II (SBP≥160); PP lower tertile = Pulse pressure 48–68 mmHg, PP middle tertile = Pulse pressure 71–77 mmHg, and PP upper tertile = Pulse pressure 84–108 mmHg.

Statistics = Chi-square test was used for categorical and ANOVA with pairwise comparisons for continuous variables.

Hx HTN = history of high blood pressure, Hx stroke = history of stroke, Hx MI = history of myocardial infarction, Hx Angina = history of angina, Hx CHF = history of congestive heart failure, Hx PAD = history of peripheral artery disease, Hx DM = history of diabetes mellitus; BMI = Body Mass Index, GDS = Geriatric Depression Scale.

**Table 2:** Means (SD) of Performances of Global and Domain Specific Cognitive Function for Exam 1–6 (9-year interval).

	Exam 1	Exam 2	Exam 3	Exam 4	Exam 5	Exam 6
	Mean (SD)					
MMSE	28.6 (1.4)	28.4 (1.8)	27.9 (2.1)	28.1 (2.5)	27.9 (2.4)	27.4 (3.0)
TMT, Part A	40.3 (12.2)	45.0 (22.0)	47.1 (24.9)	52.1 (29.1)	53.1 (33.5)	55.5 (30.6)
TMT, Part B	104.1 (35.7)	119.1 (61.1)	140.0 (80.7)	161.1 (95.2)	174.7 (104.3)	179.8 (103.2)
HVLT-I	24.2 (4.1)	23.5 (4.7)	24.3 (5.2)	22.4 (5.6)	21.8 (5.5)	22.7 (6.4)
HVLT- D	9.1 (1.9)	8.6 (2.4)	8.7 (2.5)	7.8 (2.9)	7.7 (3.1)	7.5 (3.4)

MMSE = Mini Mental State Exam; TMT, Part A = Trail Making Test, Part A; TMT, Part B = Trail Making Test, Part B; HVLT = Hopkins Verbal Learning Test (HVLT-I = immediate recall; HVLT-D = delayed recall).

The participants in the HTN II group had higher prevalence of an HTN history and reported antihypertensive medication use, and higher SBP, DBP and PP, compared to the control group. The participants in the upper tertile PP group were more often white, had lower education levels, had a higher prevalence of HTN history, MI history, CHF history, reported antihypertensive medication use, and higher SBP, DBP and PP compared to the control group (Table 1).

### SBP and Cognitive Function

In the cross sectional analyses, participants with HTN I had lower odds of impairment on the TMT, Part A (HR = 0.29 [95%CI = 0.09, 0.96]) compared to the control group. There were no differences in HTN I and II groups, and the control group in odds of impairment on the MMSE, TMT Part B, HVLT-I, HVLT-D after full adjustment in Model 2 (Table 3).

**Table 3:** Cross-Sectional Analysis of Hypertension Stages and Pulse Pressure Tertiles on Cognitive Function at Baseline.

	HTN I	HTN II	PP Middle Tertile	PP Upper Tertile
	HR (95%CI)	HR (95%CI)	HR(95%CI)	HR (95%CI)
**HVLT-D**				
Unadjusted	0.75(0.32,1.76)	0.92(0.40,2.12)	0.74(0.31,1.76)	0.94(0.42,2.10)
Model 1	0.75(0.31,1.80)	1.08(0.45,2.58)	0.80(0.32,1.96)	1.19(0.51,2.77)
Model 2	0.75(0.28,1.99)	1.37(0.51,3.70)	0.71(0.27,1.87)	1.44(0.56,3.67)
**HVLT-I**				
Unadjusted	0.96(0.44,2.10)	1.24(0.58,2.65)	0.65(0.30,1.42)	0.85(0.41,1.75)
Model 1	0.96(0.42,2.16)	1.41(0.63,3.15)	0.65(0.29,1.48)	1.00(0.47,2.14)
Model 2	0.77(0.30,1.97)	1.59(0.62,4.07)	0.52(0.21,1.28)	0.98(0.42,2.31)
**TMT, Part A**				
Unadjusted	0.45(0.18,1.11)	0.43(0.17,1.11)	0.37(0.13,1.04)	0.56(0.23,1.37)
Model 1	0.35(0.12,0.98)*	0.59(0.21,1.67)	0.44(0.14,1.38)	0.92(0.33,2.53)
Model 2	0.29(0.09,0.96)*	0.49(0.13,1.79)	0.36(0.09,1.38)	0.95(0.30,2.98)
**TMT, Part B**				
Unadjusted	1.42(0.60,3.39)	1.65(0.69,3.95)	0.74(0.31,1.80)	1.28(0.59,2.79)
Model 1	1.07(0.43,2.72)	1.56(0.62,3.90)	0.65(0.25,1.67)	1.36(0.60,3.12)
Model 2	1.26(0.42,3.73)	2.12(0.71,6.35)	0.75(0.26,2.16)	2.09(0.82,5.35)
**MMSE**				
Unadjusted	0.20(0.02,1.85)	0.69(0.15,3.12)	0.23(0.03,1.98)	0.43(0.08,2.26)
Model 1	0.15(0.01,1.45)	0.80(0.16,4.06)	0.24(0.03,2.16)	0.45(0.08,2.48)
Model 2	0.08(0.01,1.05)	0.24(0.03,2.09)	0.18(0.01,2.32)	0.25(0.03,2.29)

Reference group for HTN: control group (SBP≤139 mmHg); for PP: pulse pressure lower tertile (48–68 mmHg).

HTN I = Hypertension I (SBP 140–159 mmHg); HTN II = Hypertension II (SBP≥160); PP middle tertile = Pulse pressure 71–77 mmHg, and PP upper tertile = Pulse pressure 84–108 mmHg.

Model 1: age, race, education.

Model 2: age, race, education, history of high blood pressure, stroke, myocardial infarction, congestive heart failure, peripheral artery disease, diabetes mellitus, angina; history of smoking, BMI, ever on hypertensive medication, glucose, cholesterol, and depression score.

Note: All Trails B models adjust for Trails A performance.

MMSE = Mini Mental State Exam; TMT, Part A = Trail Making Test, Part A; TMT, Part B = Trail Making Test, Part B; HVLT = Hopkins Verbal Learning Test (HVLT-I = immediate recall; HVLT-D = delayed recall);

*p-value<0.05.

** p-value<0.01.

In the longitudinal analyses risk of cognitive impairment on any test did not differ by SBP levels (Table 4). However, when participants were stratified according to their age, participants aged between 76 and 80 years in the HTN II group had a five-fold greater increased risk of impairment on TMT-B compared to the control group (HR = 5.05 [95%CI = 1.42, 18.04]). There were no associations between SBP levels and risk of cognitive impairment among those aged 70–75 (Table 5).

**Table 4:** Longitudinal Analysis of Hypertension Stages and Pulse Pressure Tertiles on Cognitive Decline.

	HTN I	HTN II	PP Middle Tertile	PP Upper Tertile
	HR (95% CI)	HR (95% CI)	HR (95% CI)	HR (95% CI)
**HVLT-D**				
Unadjusted	1.18 (0.70–2.00)	1.02 (0.58–1.78)	0.97 (0.57–1.65)	1.06 (0.63–1.80)
Model 1	1.06 (0.62–1.81)	0.96 (0.54–1.68)	0.97 (0.56–1.66)	1.13 (0.65–1.96)
Model 2	1.23 (0.67–2.27)	1.16 (0.60–2.23)	0.88 (0.50–1.55)	1.24 (0.69–2.22)
**HVLT-I**				
Unadjusted	1.12 (0.67–1.87)	1.05 (0.61–1.80)	1.56 (0.92–2.62)	1.27 (0.74–2.18)
Model 1	1.08 (0.64–1.81)	1.03 (0.59–1.78)	1.74 (1.01–2.99)*	1.45 (0.82–2.58)
Model 2	1.35 (0.75–2.41)	1.37 (0.73–2.55)	1.53 (0.88–2.68)	1.58 (0.87–2.90)
**TMT, Part A**				
Unadjusted	1.49 (0.81–2.75)	1.34 (0.70–2.56)	0.87 (0.47–1.61)	1.11 (0.62–1.98)
Model 1	1.34 (0.71–2.50)	1.43 (0.74–2.75)	1.04 (0.55–1.95)	1.59 (0.85–2.97)
Model 2	1.44 (0.69–2.99)	1.60 (0.75–3.45)	0.93 (0.46–1.89)	1.49 (0.75–2.93)
**TMT, Part B**				
Unadjusted	1.12 (0.67–1.88)	1.20 (0.72–2.01)	0.88 (0.55–1.43)	1.46 (0.95–2.26)
Model 1	1.03 (0.61–1.73)	1.15 (0.68–1.93)	1.02 (0.62–1.69)	1.88 (1.16–3.02)
Model 2	0.90 (0.49–1.65)	1.27 (0.70–2.32)	0.98 (0.58–1.64)	2.14 (1.29–3.57)**
**MMSE**				
Unadjusted	0.90 (0.42–1.91)	1.82 (0.92–3.60)	0.96 (0.48–1.95)	1.28 (0.66–2.48)
Model 1	0.80 (0.37–1.71)	1.84 (0.92–3.67)	1.15 (0.56–2.36)	1.54 (0.75–3.13)
Model 2	0.83 (0.36–1.97)	1.93 (0.86–4.36)	1.11 (0.53–2.35)	1.27 (0.59–2.74)

Reference group for HTN: control group (SBP≤139 mmHg); for PP: pulse pressure lower tertile (48–68 mmHg).

HTN I = Hypertension I (SBP 140–159 mmHg); HTN II = Hypertension II (SBP≥160); PP middle tertile = Pulse pressure 71–77 mmHg, and PP upper tertile = Pulse pressure 84–108 mmHg.

Model 1: age, race, education.

Model 2: age, race, education, history of high blood pressure, stroke, myocardial infarction, congestive heart failure, peripheral artery disease, diabetes mellitus, angina; history of smoking, BMI, ever on hypertensive medication, glucose, cholesterol, and depression score.

Note: All Trails B models adjust for Trails A performance.

MMSE = Mini Mental State Exam; TMT, Part A = Trail Making Test, Part A; TMT, Part B = Trail Making Test, Part B; HVLT = Hopkins Verbal Learning Test (HVLT-I = immediate recall; HVLT-D = delayed recall);

*p-value<0.05.

**p-value<0.01.

**Table 5:** Age-stratified Longitudinal Analysis of Hypertension Stages and Pulse Pressure Tertiles on Cognitive Decline.

	Ages 70–75 (n = 248)			
	HTN I	HTN II	PP Middle Tertile	PP Upper Tertile
	HR (95% CI)	HR (95% CI)	HR (95% CI)	HR (95% CI)
**HVLT-D**				
Unadjusted	1.46 (0.74–2.87)	1.05 (0.49–2.27)	1.06 (0.54–2.07)	1.08 (0.55–2.11)
Model 1	1.40 (0.71–2.76)	0.91 (0.42–1.99)	1.15 (0.58–2.31)	1.25 (0.61–2.60)
Model 2	1.39 (0.68–2.84)	0.88 (0.39–2.02)	0.99 (0.47–2.09)	1.32 (0.63–2.78)
**HVLT-I**				
Unadjusted	1.57 (0.78–3.13)	1.48 (0.70–3.14)	1.92 (0.96–3.87)	1.77 (0.87–3.58)
Model 1	1.52 (0.76–3.03)	1.28 (0.60–2.72)	2.58 (1.24–5.36)**	2.65 (1.21–5.80)*
Model 2	1.47 (0.71–3.02)	1.21 (0.55–2.66)	2.44 (1.11–5.39)*	3.13 (1.35–7.24)**
**TMT, Part A**				
Unadjusted	2.52 (1.07–5.89)*	1.25 (0.45–3.46)	0.78 (0.35–1.73)	0.93 (0.43–1.99)
Model 1	2.38 (1.01–5.57)*	1.08 (0.39–3.00)	1.06 (0.47–2.39)	1.86 (0.81–4.26)
Model 2	2.13 (0.84–5.42)	1.02 (0.35–3.00)	1.01 (0.38–2.68)	1.46 (0.57–3.74)
**TMT, Part B**				
Unadjusted	1.15 (0.61–2.16)	1.05 (0.53–2.07)	0.64 (0.31–1.34)	1.71 (0.95–3.07)
Model 1	1.09 (0.58–2.07)	0.87 (0.43–1.74)	0.68 (0.32–1.45)	1.83 (0.96–3.49)
Model 2	1.01 (0.51–1.99)	1.04 (0.50–2.16)	0.58 (0.25–1.32)	1.83 (0.91–3.71)
**MMSE**				
Unadjusted	0.65 (0.25–1.70)	1.43 (0.60–3.41)	0.97 (0.40–2.33)	0.98 (0.41–2.37)
Model 1	0.56 (0.22–1.47)	1.02 (0.43–2.46)	1.02 (0.42–2.49)	1.00 (0.38–2.60)
Model 2	0.47 (0.17–1.30)	1.07 (0.41–2.80)	1.03 (0.39–2.72)	0.97 (0.34–2.73)

	Ages 76–80 (n = 88)			
	HTN I	HTN II	PP Middle Tertile	PP Upper Tertile
	HR (95% CI)	HR (95% CI)	HR (95% CI)	HR (95% CI)
**HVLT-D**				
Unadjusted	0.93 (0.39–2.25)	0.88 (0.39–2.00)	0.65 (0.27–1.55)	0.79 (0.34–1.83)
Model 1	0.85 (0.35–2.08)	0.92 (0.40–2.08)	0.64 (0.26–1.56)	0.79 (0.33–1.90)
Model 2	0.82 (0.27–2.47)	1.13 (0.38–3.43)	0.80 (0.27–2.31)	2.08 (0.54–7.98)
**HVLT-I**				
Unadjusted	0.85 (0.38–1.91)	0.59 (0.27–1.30)	0.82 (0.37–1.81)	0.51 (0.21–1.20)
Model 1	0.81 (0.36–1.84)	0.61 (0.28–1.35)	0.80 (0.36–1.79)	0.47 (0.19–1.16)
Model 2	0.96 (0.38–2.45)	0.98 (0.37–2.59)	0.68 (0.27–1.71)	0.86 (0.25–2.93)
**TMT, Part A**				
Unadjusted	0.68 (0.24–1.93)	1.15 (0.49–2.69)	0.77 (0.29–2.06)	1.03 (0.40–2.61)
Model 1	0.65 (0.23–1.85)	1.18 (0.50–2.76)	0.81 (0.29–2.24)	1.08 (0.40–2.94)
Model 2	0.80 (0.22–2.97)	2.56 (0.73–9.06)	0.52 (0.15–1.87)	2.01 (0.49–8.28)
**TMT, Part B**				
Unadjusted	1.26 (0.52–3.03)	1.30 (0.59–2.88)	0.75 (0.31–1.79)	0.95 (0.41–2.18)
Model 1	1.20 (0.50–2.89)	1.39 (0.62–3.11)	0.74 (0.30–1.81)	1.05 (0.42–2.65)
Model 2	2.13 (0.65–6.95)	5.05 (1.42–18.04)*	1.24 (0.36–4.28)	5.12 (1.11–23.62)*
**MMSE**				
Unadjusted	-^#^	-^#^	0.74 (0.23–2.44)	1.37 (0.47–4.02)
Model 1	-^#^	-^#^	1.11 (0.30–4.09)	1.89 (0.58–6.19)
Model 2	-^#^	-^#^	0.83 (0.20–3.47)	2.36 (0.40–13.83)

Reference group for HTN: Control group (SBP≤139 mmHg): for PP: pulse pressure lower tertile (48–68 mmHg).

HTN Stage I = Hypertension Stage I (SBP 140–159 mmHg); HTN Stage II = Hypertension Satge II (SBP≥160); PP middle tertile = Pulse pressure 71–77 mmHg, and PP upper tertile = Pulse pressure 84–108 mmHg.

Model 1: age, race, education.

Model 2: age, race, education, history of high blood pressure, stroke, myocardial infarction, congestive heart failure, peripheral artery disease, diabetes mellitus, angina; history of smoking, BMI, ever on hypertensive medication, glucose, cholesterol, and depression score.

Note: All Trails B models adjust for Trails A performance.

MMSE = Mini Mental State Exam; TMT, Part A = Trail Making Test, Part A; TMT, Part B = Trail Making Test, Part B; HVLT = Hopkins Verbal Learning Test (HVLT-I = immediate recall; HVLT-D = delayed recall);

*p-value<0.05.

**p-value<0.01.

### PP and Cognitive Function

In cross sectional analyses odds of impairment on any cognitive test did not differ by PP tertile (Table 3).

In the longitudinal analyses, participants in the upper versus lower tertile, had a greater risk of impairment in executive function assessed by TMT, Part B (HR = 2.14 [95%CI = 1.29, 3.57]) compared to the lower tertile. Notably, this association was largely driven by the women aged 76–80 years old in the highest PP tertile who had a five-fold greater risk of TMT, Part B impairment compared to the lower tertile (HR = 5.12 [95%CI = 1.11; 23.62]) (Table 3 and 4). However, participants aged between 70–75 years in both the middle and upper tertile group had a greater risk of verbal learning impairment assessed by HVLT-I (HR = 2.44, 95% CI [1.11, 5.39]; HR = 3.13) [95%CI = 1.35, 7.24], respectively (Table 4) when compared to the control group.

## Discussion

In this study we evaluated effects of elevated SBP or PP on cognitive abilities, such as global cognitive function, speed of processing, executive function, visuospatial function, and verbal learning and memory, at baseline and over a 9-year period in non-demented older community-dwelling female participants, in the WHAS II. Our results showed that women aged between 76–80 years with SBP≥160 mmHg or with PP≥84 mmHg showed five times higher incidence of impairment on TMT, Part B, a measure of executive function, when compared to their control groups. We also found that participants aged 70–75 years at baseline with PP≥84 mmHg had two- to three-fold higher incidence of impairment in HVLT-I, a measure of verbal learning. Our data suggest that elevated SBP and PP in older non-demented women increases the risk for cognitive impairment in late-life.

With increasing life expectancy the number of older people, especially older women who have a higher prevalence of HTN [3], including elevated SBP and/or DBP, also increases. HTN in midlife has been shown to be risk factor for lower cognitive function in later life [7], but there is mixed evidence for an effect of HTN in late-life on cognitive function, with information mostly limited to measures of global cognitive function [14]–[18], [37]. There are few studies, with none looking specifically at older women, reporting associations between elevated SBP and impairment in specific cognitive abilities in late life [13], [20], [21], and findings from these studies indicate that the affected cognitive abilities are dependent on the participants' age and the length of follow-up [19]–[21]. Our results extend these findings to older women, aged between 76–80 years, by showing detrimental effects of elevated SBP≥160 mmHg and PP≥84 mmHg in late life on executive function, in the absence of dementia, over a 9-year period. Our study also suggests that PP is maybe a more sensitive measure for cognitive decline, since it takes into account both the effects of SBP and DBP.

Several mechanisms have been proposed and evaluated to explain associations between elevated blood pressure and specific cognitive abilities. It has been suggested that chronic HTN, by hastening the atherosclerotic process in deep white matter vessels, has a detrimental effect on sub-cortical white matter circuits [7], which may account for impairment in specific cognitive abilities such as executive function [38]. This seems to be confirmed by imaging studies using magnetic resonance imaging (MRI) of individuals with HTN which have found an increased number of white matter abnormalities in the frontal lobe [39]. Our current findings are in line with these studies, namely we found an association between elevated SBP and impairment on TMT, Part B, a measure of executive function.

There is a long standing hypothesis that HTN-associated changes in the brain may interact with age-associated changes. Thus the measured effect will be larger in older than in younger hypertensive people [40] and this effect could be captured better by measures of arterial stiffness such as PP rather than SBP. Our study partially supports this hypothesis by showing the detrimental effect of elevated SBP and also PP only in women aged 76–80, but not those aged 70–75. However, verbal learning was found to be worse in the younger group with the highest PP and this effect was not seen in the older group and also not seen when evaluating the effect of SBP. This could be partially explained by the different brain areas involved in the different tasks, i.e. executive function versus verbal learning, and their sensitivity to hypertension- and age-associated changes.

There are some limitations of this study. First, the sample size was relatively small and was composed of women, which may limit generalization of these findings to the whole population. Second, as is true in all observational studies, our results may be vulnerable to confounding. It is possible that HTN and risk of cognitive decline reflect the association of antihypertensive medication use with yet another unmeasured variable. Third, we conducted numerous comparisons, which could raise the possibility of our significant findings being there by chance. Fourth, information on mild cognitive impairment or dementia diagnosis is not currently available, but adjudication is ongoing. Fifth, as in all studies with aging cohorts, survival bias may be an issue, since people with HTN might be more likely to die due to the increased mortality risk associated with HTN. Last, there is selective loss to follow-up of those most impaired, as found in most population-based studies, but this would lead to underestimation of true rates of decline and a more conservative estimate of our findings.

There are a number of advantages of this study. First, is its population-based study which examined only female participants initially free of cognitive and functional impairment and followed them up to 9 years. Second, there were frequent and repeated comprehensive cognitive assessments. Third, we were able to take into consideration the effects of antihypertensive medications and their effects on the brain and cognitive function by adjusting for antihypertensive medication use.

Moderately elevated SBP levels have been considered acceptable in older patients due to fear of hypoperfusion. However, clinical trials, and most recently the Hypertension in the very Elderly (HYVET) clinical trial [41], have shown that reducing blood pressures in people older than 80 years from 173/91 to at least 150/80 mmHg in 48% of the people in the active treatment group resulted in decreased death from stroke and death from any-cause. Although, it needs to be noted that the HYVET-Cog study showed no benefits in terms of prevention of dementia or incidence of global cognitive decline [42], which may have been due to either the short follow-up secondary to the early termination of the trial, or to the lack of treatment effect. Although our study was an observational study, our results could provide additional argument that treating elevated SBP≥160 mmHg in the older population, especially women, could not only result in decreased mortality, but also in the preservation of cognitive function, specifically executive function.

Our data suggests that elevated SBP or PP in older non-demented women increases risk for late-life cognitive impairment. These results warrant further, larger studies to evaluate possible effects of elevated blood pressure in normal cognitive aging.
